# Differential Angiogenic Properties of Lithium Chloride *In Vitro* and *In Vivo*

**DOI:** 10.1371/journal.pone.0095546

**Published:** 2014-04-21

**Authors:** Ludwig F. Zeilbeck, Birgit Müller, Verena Knobloch, Ernst R. Tamm, Andreas Ohlmann

**Affiliations:** Institute of Human Anatomy and Embryology, University of Regensburg, Regensburg, Germany; University of California Davis, United States of America

## Abstract

Wnt/β-catenin signaling induced by the Norrin/Frizzled-4 pathway has been shown to improve capillary repair following oxygen induced retinopathy (OIR) in the mouse, a model for retinopathy of prematurity. Here we investigated if treatment with the monovalent cation lithium that has been shown to augment Wnt/β-catenin signaling *in vitro* and *in vivo* has similar effects. In cultured human microvascular endothelial cells, LiCl as well as SB 216763, another small molecule that activates Wnt/β-catenin signaling, induced proliferation, survival and migration, which are all common parameters for angiogenic properties *in vitro*. Moreover, treatment with both agents caused an increase in the levels of β-catenin and their translocation to nuclei while quercetin, an inhibitor of Wnt/β-catenin signaling, completely blocked the effects of LiCl on proliferation. In mice with OIR, intraperitonal or intravitreal treatment with LiCl markedly increased the retinal levels of β-catenin, but did not improve capillary repair. In contrast, repair was significantly improved following intravitreal treatment with Norrin. The effects of LiCl on HDMEC *in vitro* have minor relevance for OIR *in vivo*, and the influence of the Norrin/Frizzled-4 pathway on capillary repair in OIR is not reproducible upon enhancing Wnt/β-catenin signaling by LiCl treatment strongly indicating the presence of additional and essential mechanisms.

## Introduction

Ischemic retinal diseases, such as diabetic retinopathy or retinopathy of prematurity, are among the leading cause of blindness in developed countries [1]. Retinal ischemia is caused by lack or loss of capillaries, a scenario which activates the typical hypoxia response leading to an increase in the expression of angiogenic molecules such as vascular endothelial growth factor (VEGF). The increase in angiogenic molecules does not lead to a restoration of the normal retinal capillary bed, but rather causes retinal neovascularization, a condition in which newly formed vessels grow along the retina and into the vitreous. Vitreous hemorrhages or fluid exudation from newly formed or existing vessels are frequent complications. VEGF is the factor which is primarily involved in vascular proliferation during retinal neovascularization and the increase in vascular permeability [2], [3], [4], [5]. Current therapeutic options such as photocoagulation or applying antibodies against VEGF aim at improving oxygen supply to the inner retina or at blocking the effects of VEGF [5], [6]. So far, no treatment is available that promotes regrowth of functional capillaries into ischemic retinal areas.

During development, the formation of retinal capillaries depends on signaling pathways that differ between the different capillary beds of the retina. The initial outgrowth of capillaries on the retinal surfaces is largely driven by VEGF which is mainly released from retinal astrocytes [7], [8]. In contrast, capillary formation in the deep vascular plexus of the retina depends on an essential signaling system involving the secreted signaling molecule Norrin, its receptor Frizzled-4, and the activation of canonical Wnt/β-catenin signaling [9], [10], [11], [12]. Accordingly, Norrin or Frizzled-4-deficient mice develop capillaries on the retinal surface, but lack intraretinal capillaries [13], [14], [15]. In previous work, we showed that the transgenic overexpression of Norrin rescues the phenotype of Norrin-deficient mice without causing retinal neovascularization [13]. Moreover, Norrin protected mice against oxygen-induced vascular loss after oxygen-induced retinopathy (OIR), an animal model for retinopathy of prematurity [16]. In mice, OIR is generated by an exposure to 75% oxygen from postnatal day (P)7 to P12 to induce an obliteration of the central retinal vasculature [17]. After oxygen exposure, vessels regrow into vaso-obliterated areas and preretinal neovascular tufts form [17]. After induction of an OIR in mice with a transgenic overexpression of Norrin in the lens, Norrin enhances vessel regrowth into vaso-obliterated areas und promotes the formation of intraretinal vessels [16]. This effect was substantially diminished in mice with an additional treatment of DKK-1, an inhibitor of Wnt/β-catenin signaling [16]. In addition, the number of preretinal neovascular tufts was markedly reduced [16]. In human patients, the formation of preretinal neovascular tufts is a severe complication of retinopathy of prematurity or diabetic retinopathy.

Since the protective effects of Norrin in OIR required the activity of canonical Wnt/β-catenin signaling, we wondered if this pathway has the potential to be used for capillary regrowth and/or maintenance in the treatment of retinal ischemic diseases. A molecule that activates canonical Wnt/β-catenin signaling in the mouse brain upon oral administration [18] is the monovalent cation lithium which has been used in the treatment of bipolar disorders for decades [19], [20]. Quite intriguingly, delayed chronic lithium treatment in rats suffering from stroke after permanent middle cerebral artery occlusion resulted in enhanced blood oxygenation levels indicating a positive effect on vascular formation in poststroke animals [21]. Moreover, in a rat model of myocardial infarction, lithium caused an accumulation of β-catenin in parallel to a positive effect on capillary density [22]. Lithium has been shown to activate canonical Wnt/β-catenin signaling *in vitro* and *in vivo*, an effect which is thought to involve the inhibition of the enzyme glycogen synthase kinase-3β (GSK-3β) which decreases the cytoplasmic β-catenin levels through phosphorylation-dependent proteasomal degradation [18], [23], [24], [25]. In the present study, we investigated if activation of canonical Wnt/β-catenin signaling following treatment with LiCl induces angiogenic properties in cultured microvascular endothelial cells and improves capillary recovery following OIR in mice.

## Materials and Methods

### Animals

All procedures in this study conformed to the Policies on the Use of Animals and Humans in Neuroscience Research of the Society for Neurosciences, and were approved by the local authority (Regierung der Oberpfalz, Bavaria, Germany; permit number: 54-2532.1-09/11). All surgery was performed under isofluorane anesthesia, and all efforts were made to minimize suffering. Mice were housed under standard laboratory conditions (12–12 h light-dark cycle, lights on at 6 am, 22°C, and 60% humidity) with food and water *ad libitum*.

### Oxygen-induced Retinal Damage

At postnatal day (P) 7, FVB/n mice including their mother were exposed to 75% oxygen for 5 days [17]. After oxygen exposure at P12, mice were anesthetized with isofluorane (Baxter) and injected intraperitoneally (i.p.) with LiCl (50 µg/g body weight; Merck) or phosphate buffered saline (PBS), or treated with an intravitreal injection of LiCl (10 µg) in one eye whereas the fellow eye received PBS only. For protein analyses, eyes were enucleated 6 h after LiCl treatment and dissected retinae were subjected to protein isolation and western blot analyses. In parallel experiments, mice were either injected with LiCl (i.p., 50 µg/g body weight) every 12 h from P12 to P14 or intravitreally injected once with LiCl (10 µg), Norrin (10 ng/µl) or 3 µl SB 216763 (5 µg/µl) after oxygen exposure. To analyze the effects of LiCl on capillary regrowth into vaso-obliterated areas at P13 (intravitreal injection) or P14 (i.p. injection), mice were perfused with FITC-dextran as previously described [16]. In brief, mice were perfused with FITC-dextran (TdB Consultancy, Uppsala, Sweden), retinal whole mounts were isolated and analyzed on a fluorescent microscope (Axiovision, Carl Zeiss). The total area of the superficial vascular plexus and the area of vaso-obliteration were measured and calculated as relative area of vaso-obliteration per total area of the superficial vascular plexus using the Axiovision software 4.8.2 (Carl Zeiss).

### Cell Culture, Proliferation, Viability, Migration

Human dermal microvascular endothelial cells (HDMEC) were purchased from Promocell (Heidelberg, Germany) and cultured in supplemented Microvascular Endothelial Cell Growth Medium (Promocell) containing penicillin (100 U/ml) and streptomycin (100 g/ml) at 37°C and 5% CO_2_. For all experiments unsupplemented cell culture medium was used. Confluent HDMEC were incubated for 3 h with LiCl (0.2 mM, 1 mM, 10 mM; Merck) or SB 216763 (5 µM, 10 µM, 20 µM dissolved in DMSO; Sigma) in unsupplemented cell culture medium and subjected to western blot analyses or immunocytochemistry (see below).

Cell proliferation assays were performed by BrdU labeling of dividing cells according to the manufacturer’s instructions (Roche). In brief, 4000 HDMEC were seeded in 96-well tissue culture plates, allowed to attach overnight and incubated with an unsupplemented cell culture medium that contained BrdU (10 µM) and LiCl (0.2 mM, 1 mM, 10 mM) with or without quercetin (10 µM dissolved in DMSO; Sigma), or BrdU (10 µM) and SB 216763 (5 µM, 10 µM, 20 µM; dissolved in DMSO). In addition, control cells were treated with 0.2 or 1 mM NaCl, 50 ng/ml VEGF (Biolegend, London, UK) or DMSO. After 24 h, cells were fixed and BrdU incorporation was measured by ELISA at 450 nm (Tecan, Crailsheim, Germany). Cell viability was investigated by measuring the NADH- and NADPH-dependent turnover of WST-1 dye according to the manufacturer’s instructions (Roche). In brief, 4000 HDMEC were seeded in 96-well tissue culture plates, allowed to attach overnight and incubated in unsupplemented cell culture medium that contained LiCl (0.2 mM, 1 mM, 10 mM) or SB 216763 (5 µM, 10 µM, 20 µM). Again, additional control cells were treated with 0.2 or 1 mM NaCl, 50 ng/ml VEGF or DMSO. After 72 h, the medium was removed and unsupplemented cell culture medium containing WST-1 was added for 30 min. Extinction was detected at 450 nm on an ELISA reader (Tecan). Cell migration was quantified by standard scratch assays as described previously [16]. After starvation for 6 h without supplement, confluent HDMEC monolayers were scratched using a 200 µl sterile plastic pipette tip. Loose cells were removed, followed by an incubation of the cells with unsupplemented cell culture medium containing mitomycin C (5 g/ml; Sigma), and LiCl (0.2 mM, 1 mM), NaCl (0.2 mM, 1mM) or VEGF (50 ng/ml). Sizes of scratched areas were analyzed after 0 and 18 h of incubation using a phase contrast microscope (Axiovert M400, Carl Zeiss). Cell migration was calculated as relative recolonized area (µm^2^) per length of the scratch (µm) by using the Axiovision software 4.8.2 (Carl Zeiss).

### VEGF ELISA

VEGF ELISA was purchased from Biorbyt (Cambridge, UK) and was performed in accordance with the manufacturer’s instructions. In brief, 10.000 HDMEC were cultured on a 96-well cell culture dish, allowed to attach, starved overnight in unsupplemented cell culture medium and incubated in unsupplemented cell culture medium with 0.2 mM NaCl or 0.2 mM LiCl. After 24 h, 100 µl of conditioned cell culture medium were subjected to VEGF ELISA that was measured by an ELISA plate reader at 450 nm (Tecan).

### Immunochemistry

For immunocytochemistry, HDMEC were fixed with methanol for 5 min 3 h after treatment with 1 mM LiCl or 10 µM SB 216763 in unsupplemented cell culture medium. For immunohistochemistry, LiCl-treated FVB/n mice were perfused with 4% PFA. Enucleated eyes were fixed in 4% PFA for 2 h, embedded in Tissue Tek (Sakura Finetek) according to standard protocols and subjected to cryosectioning. Prior to incubation with rabbit anti-β-catenin antibodies (1∶100; Cell Signaling Technology) overnight at 4°C, samples were blocked for 45 min with 0.1 M phosphate buffer containing 3% bovine serum albumin (BSA) and 0,1% Triton X-100. After three washes (10 min each) with 0.1 M phosphate buffer, samples were treated with biotin-labeled goat anti-rabbit antibodies (1∶500; Vector Laboratories) for 1 h. Following three additional washes, cells were incubated for 1 h with streptavidin-Alexa 488 Fluor conjugates (1∶1000; Invitrogen). Finally, specimens were washed again three times and mounted in fluorescent mounting medium containing 1∶10 DAPI (Vector Laboratories). Immunochemical staining was analyzed in the ApoTome mode of an Axiovision fluorescent microscope (Carl Zeiss).

### Protein Analyses

For western blot analyses of β-catenin levels, total protein was extracted using RIPA buffer. Homogenized mouse retinae or HDMEC were dissolved in RIPA buffer, and cellular debris was removed by centrifugation. Protein concentration was measured by the BCA (Interchim) method and up to 25 µg of total proteins were subjected to a 10% SDS-PAGE. Proteins were subsequently transferred on a PVDF membrane (Roche) by semidry blotting. After blocking with 5% low fat milk in TBS-T, the PVDF membranes were incubated overnight at 4°C with rabbit anti-β-catenin antibodies (Cell Signaling Technology), diluted 1∶1000, in TBS-T with 0.5% low fat milk. HRP-conjugated chicken anti-rabbit antibodies (Santa Cruz Biotechnology) were used as secondary antibodies at a 1∶2000 dilution in TBS-T with 0.5% low fat milk. Antibody labeling was visualized using the Immobilon HRP substrate (Millipore). For documentation a LAS 3000 Imager work station (Fujifilm) was used. HRP-conjugated anti-GAPDH antibodies (Rockland) were applied as loading control.

### Statistics

All values are expressed as mean ± SEM. Comparisons between the mean variables of 2 groups were made by a two-tailed Student’s t-test. P-values less than 0.05 were considered to be statistically significant.

## Results

### LiCl and SB 216763 Activate the Wnt/β-catenin Pathway in HDMEC

**Figure 1:**
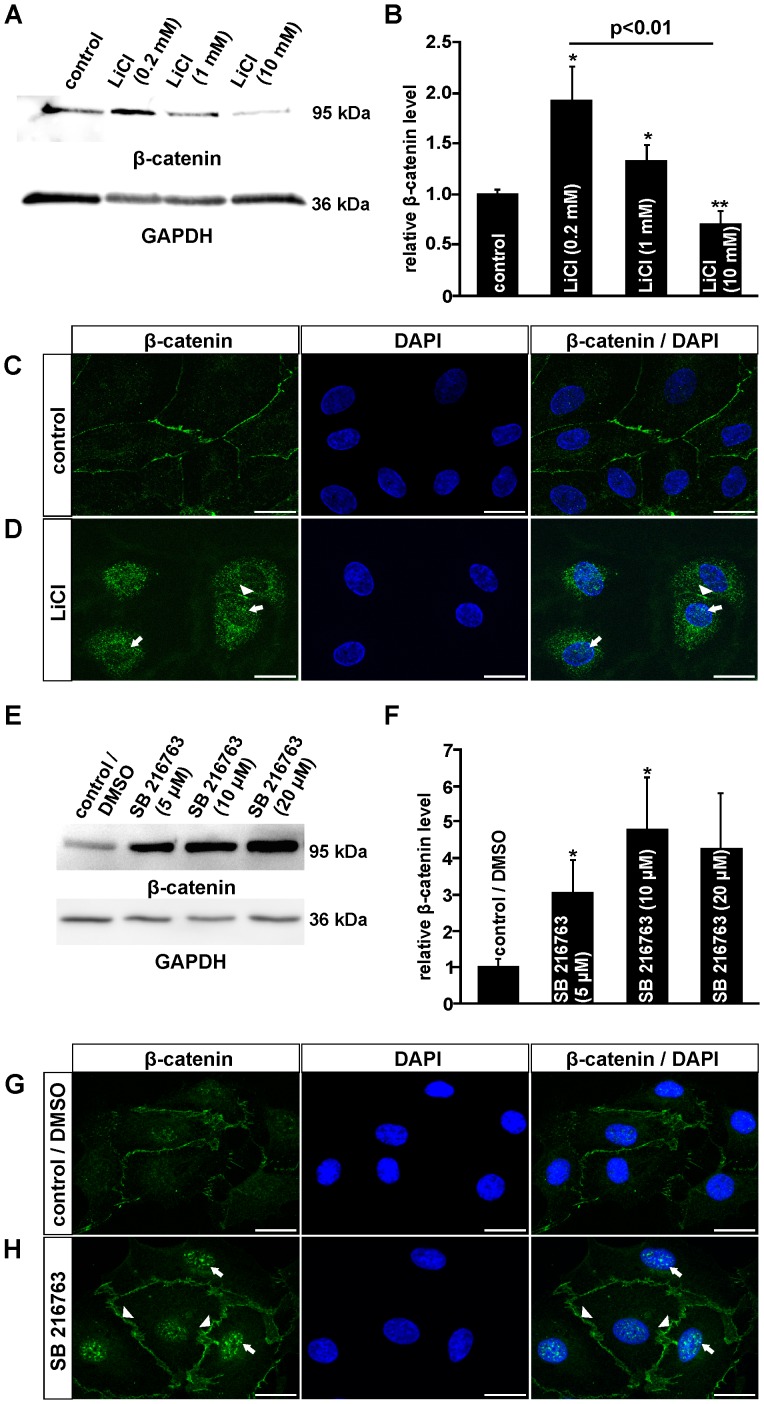
LiCl and SB 216763 activate the canonical Wnt/β-catenin pathway in HDMEC. A, B, E, F. Western Blot analyses (A, E) and relative densitometry (B, F) for β-catenin in total protein of HDMEC after treatment with LiCl (A, B) or SB 216763 (E, F) for 3 h (mean ± SEM of 5 independent experiments; n≥5; *p<0.05; **p<0.01). C, D, G, H. Immunocytochemistry for β-catenin (green) in HDMEC after incubation with 1 mM LiCl (D) or 10 µM SB 216763 (H) for 3 h and untreated controls (C, G). Blue, DAPI staining. Scale bars: 20 µm.

To analyze if LiCl or SB 216763 which similarly block the activity of GSK-3β on β-catenin degradation [26] both activate the canonical Wnt/β-catenin signaling pathway in human dermal microvascular endothelial cells (HDMEC), immunohistochemistry and western blot analyses were performed. After an incubation of HDMEC with LiCl for 3 h, specific bands for β-catenin were detected in isolated cellular proteins. By densitometry, the incubation of HDMEC with 0.2 mM or 1 mM LiCl caused an increase in β-catenin of up to 1.9- or 1.3-fold, respectively (Fig. 1A, B). In contrast, following treatment with 10 mM LiCl, a 25% decrease of β-catenin amounts was detected when compared with untreated controls (Fig. 1A, B). After incubation of HDMEC with SB 216763, a more substantial increase in the amounts of β-catenin was detected by western blot analyses (Fig. 1E). Densitometry showed a significant 3.1- or 4.8-fold increase after treatment of the cells with 5 µM or 10 µM SB 216763, respectively (Fig. 1F). To confirm that treatment of HDMEC with LiCl or SB 216763 not only induced an accumulation of cytoplasmic β-catenin but also lead to its translocation into the nucleus, immunohistochemistry for β-catenin was performed. In untreated control cells, a weak cytoplasmic signal for β-catenin was detected in addition to a moderate signal close to the cell membrane (Fig. 1C, G). After treatment of HDMEC with LiCl, the cell membrane-associated signal for β-catenin became considerably weaker (Fig. 1D, arrowheads) and a distinct signal was now observed in the perinuclear cytoplasm and the nucleus (Fig. 1D, arrow). Treatment with SB 216763 for 3 h did not change the intensity of the β-catenin signal at the cell membrane (Fig. 1G, H; arrowheads). In addition, a strong increase in nuclear β-catenin staining was observed (Fig. 1H; arrow), indicating a substantial translocation of β-catenin into the nucleus. Overall, our data strongly suggested that treatment with both LiCl and SB 216763 caused a dose-dependent activation of the Wnt/β-catenin signaling pathway. Treatment with SB 216763 appeared to have stronger effects than LiCl.

### LiCl Induces Proliferation of Microvascular Endothelial Cells *in vitro* via Wnt/β-catenin Signaling

**Figure 2:**
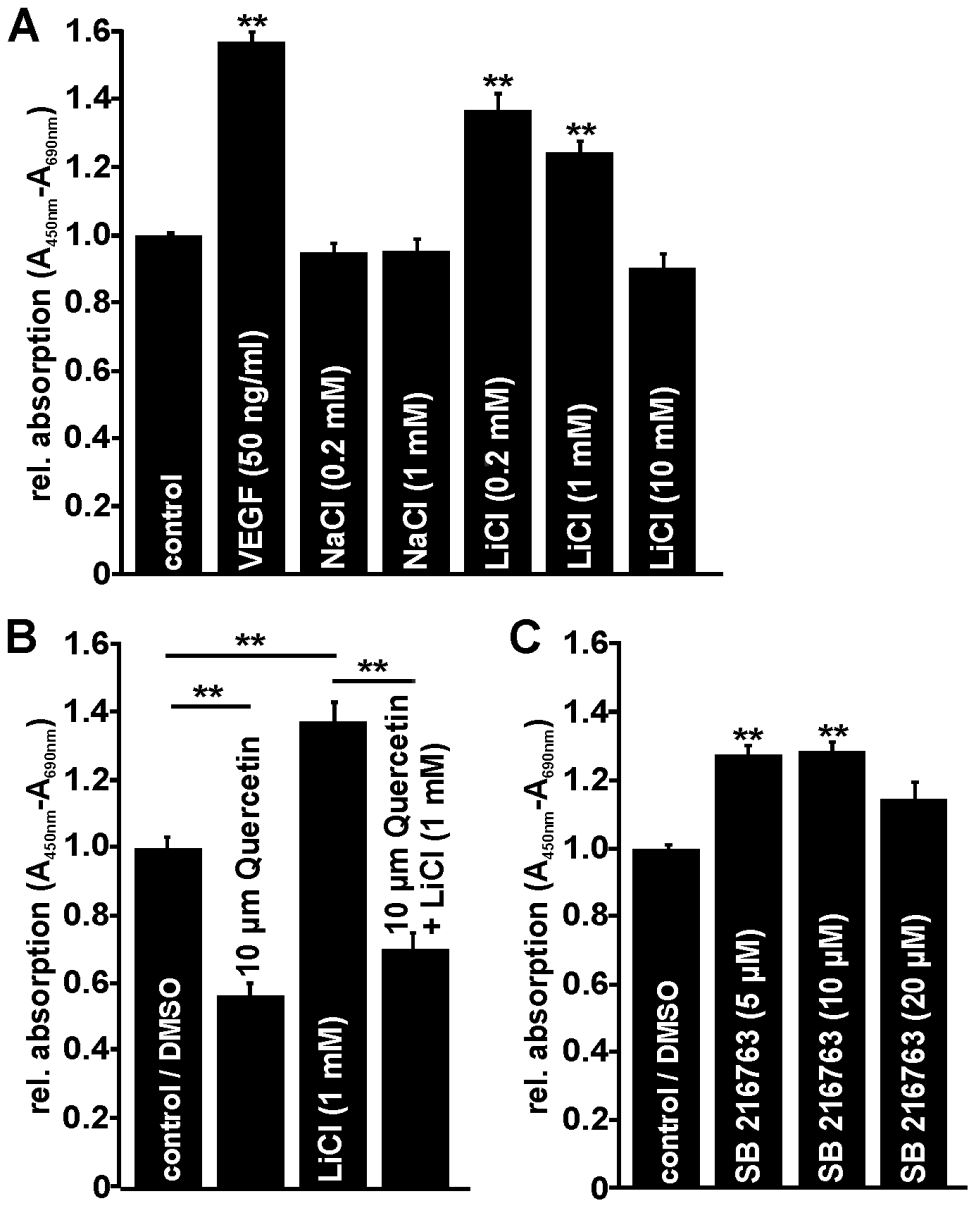
LiCl and SB 216763 induce proliferation of HDMEC *in vitro*. Proliferation of HDMEC was quantified after treatment with NaCl, LiCl and VEGF (A) or SB 216763 (C). In HDMEC, VEGF (50 ng/ml), LiCl (0.2 mM, 1 mM) and SB 216763 (5 µM, 10 µM) significantly induced cell proliferation after incubation for 24 h (mean ± SEM; n≥63 from 4 independent experiments; **p<0.01). The effect of LiCl was markedly blocked by co-incubation with 10 µM quercetin, an inhibitor of Wnt/β-catenin signaling (B, mean ± SEM; n≥24 from 3 independent experiments; **p<0.01).

To determine whether LiCl promotes angiogenic properties in microvascular endothelial cells *in vitro*, proliferation, survival and migration of HDMEC were analyzed. Proliferation of HDMEC was investigated by BrdU ELISA. In cells that were incubated with 0.2 mM or 1 mM NaCl for negative control no increase in BrdU uptake was observed (Fig. 2A). However, cultivation of HDMEC with VEGF (50 ng/ml) induced proliferation of more than 55% compared with untreated control cells (Fig. 2A). Treatment with 0.2 or 1 mM LiCl for 24 h caused a significant up to 38% increase in the amounts of incorporated BrdU when compared to untreated controls (Fig. 2A). No effect was seen after treatment of the cells with 10 mM LiCl (Fig. 2A). Intriguingly, when compared with VEGF-mediated proliferation, the effect of 0.2 mM LiCl on HDMEC was approximately 65% (Fig. 2A).

To investigate if LiCl induces HDMEC proliferation via Wnt/β-catenin signaling, cells were incubated with LiCl in the presence of 10 µM quercetin, a specific and potent inhibitor of β-catenin signaling [27]. Again, a significant proliferative effect on HDMEC was detected following LiCl-treatment. In contrast, no increase in BrdU-incorporation was observed after combined treatment with LiCl and quercetin (Fig. 2B), strongly suggesting the involvement of Wnt/β-catenin signaling in the proliferative effects of LiCl. Quite intriguingly, quercetin itself significantly decreased the proliferation of HDMEC compared with untreated control cells (Fig. 2B).

Finally, we treated HDMEC with SB 216763 to observe a significant approximately 30% increase in proliferation when added at concentrations of 5 µM or 10 µM (Fig. 2C). No effect was observed when 20 µM SB 216763 were added (Fig. 2C). Taken together our data strongly indicate that an activation of Wnt/β-catenin signaling by LiCl or SB 216763 induces proliferation of cultured HDMEC.

### LiCl Induces Survival of Microvascular Endothelial Cells *in vitro*

**Figure 3:**
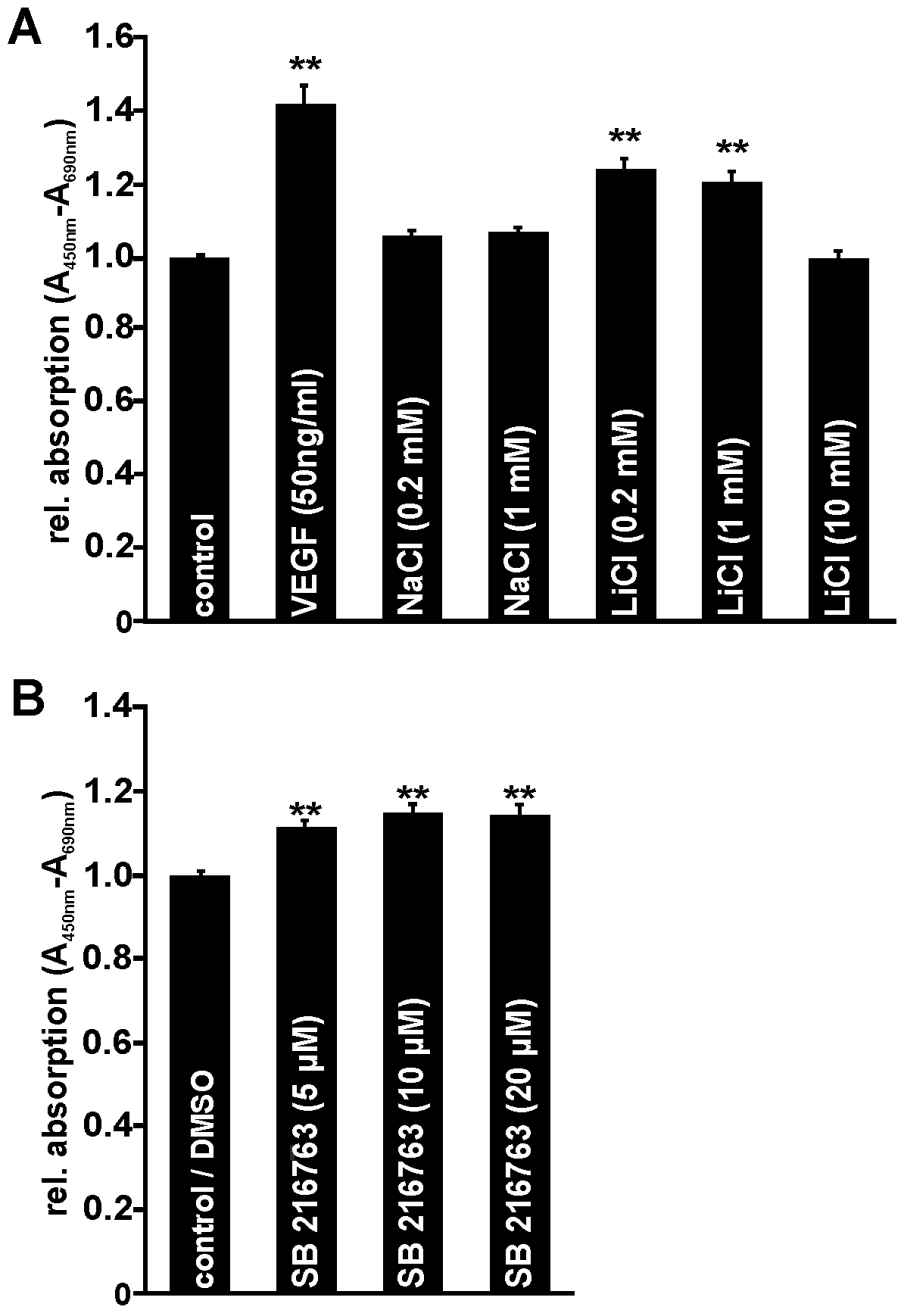
LiCl and SB 216763 promote HDMEC survival *in vitro*. Quantification of HDMEC viability after incubation with NaCl, LiCl and VEGF (A) or SB 216763 (B) for 72 h. LiCl (0.2 mM, 1 mM) and VEGF (50 ng/ml) markedly increased HDMEC survival (A) whereas SB 216763 had only a moderate positive effect on cell viability (B; mean ± SEM; n≥32 from 4 independent experiments; **p<0.01).

To analyze whether LiCl can promote their survival, HDMEC were incubated with LiCl for 72 h in unsupplemented cell culture medium. Again, incubation of 0.2 and 1 mM NaCl had no effect on microvascular endothelial cell viability (Fig. 3A). When compared with controls, treatment with 0.2 or 1 mM LiCl led to a significant 20–25% increase in cell survival (Fig. 3A). Likewise, after incubation with VEGF that was used as positive control, 42% more cells survive compared with untreated controls (Fig. 3A). However, treatment of HDMEC with a higher dose of LiCl (10 mM) had no effect on cell survival compared to control cells (Fig. 3A). Next we analyzed the effects of SB 216763 treatment on HDMEC survival. After incubation with 5, 10, or 20 µM SB 216763 for 72 h, a significant up to 15% increase in cell number was detected when compared with untreated control cells (Fig. 3B).

### LiCl Induces Migration of Microvascular Endothelial Cells *in vitro*

**Figure 4:**
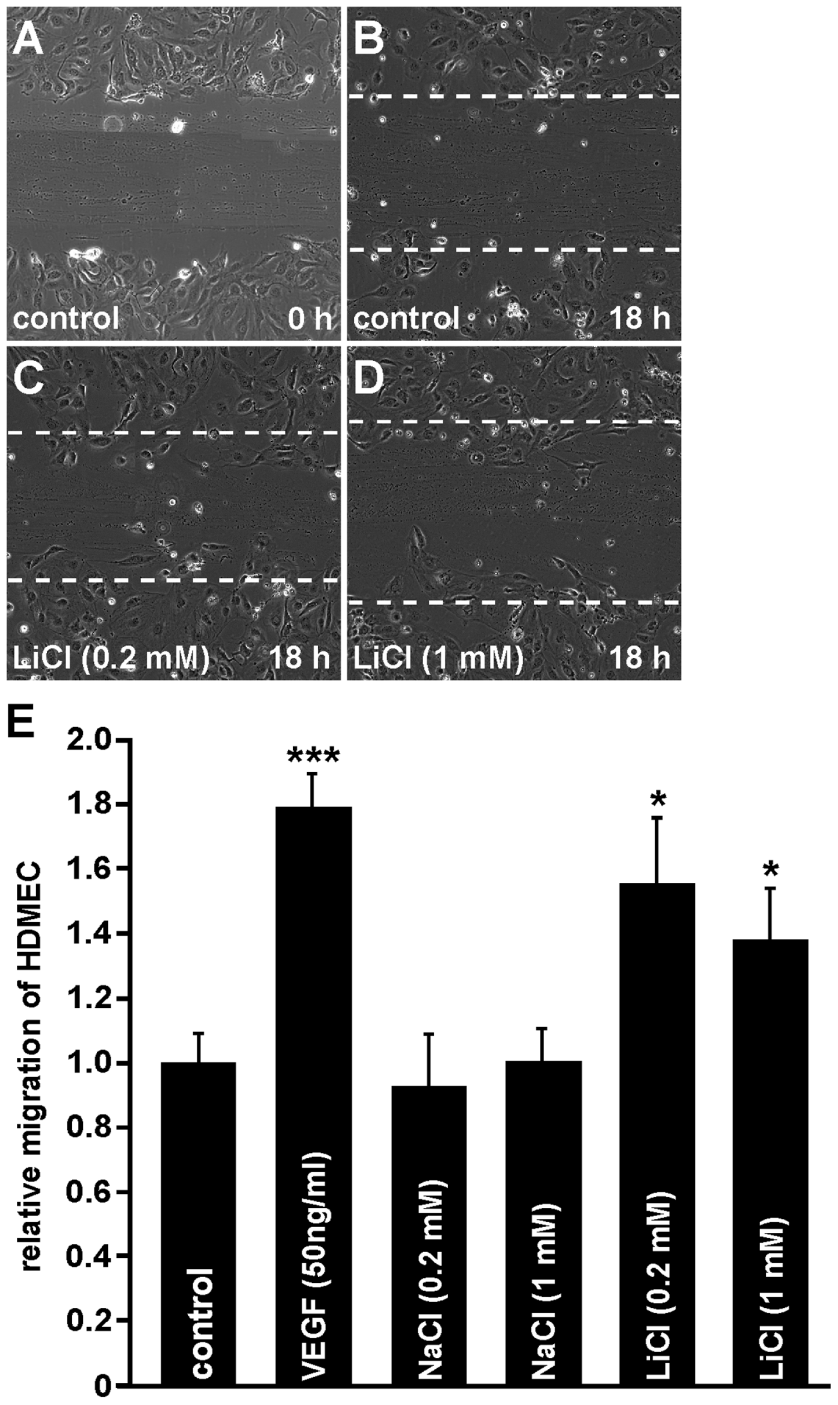
LiCl increases migration of HDMEC *in vitro*. A–E. In confluent HDMEC a scratch was performed and migration of HDMEC was calculated as recolonized area per length of the scratch after incubation with LiCl for 18 h (B–D; dashed line mark the areas lacking cells before incubation; E, mean ± SEM; n≥10 from at least 2 independent experiments; *p<0.05).

To investigate the effects of LiCl on migration of HDMEC, scratch migration assays were performed. In untreated control cells only a weak migration of cells into the scratch area was seen (Fig. 4B). Similar observations were made after incubation of HDMEC with 0.2 or 1 mM NaCl (Fig. 4E). In contrast, after incubation of HDMEC with LiCl the number of invading cells was obviously higher than in untreated controls (Fig. 4C, D). The strongest effect on HDMEC migration was seen after treatment with VEGF that was used as positive control. Quantification showed that following incubation with 0.2 or 1 mM LiCl migration of HDMEC was significantly increased by 56% and 38%, respectively (Fig. 4E), compared with untreated controls. In addition, the LiCl-induced effect (0.2 mM) on microvascular endothelial cell migration was 70% of that caused by VEGF treatment (Fig. 4E).

### LiCl Treatment dose not Induce VEGF Expression

Since there is evidence that the activation of Wnt/β-catenin signaling enhances the expression of VEGF [28], we wondered if the effects of LiCl on HDMEC proliferation, viability and migration could be mediated via an induction of VEGF. To this end, HDMEC were incubated with 0.2 mM NaCl or 0.2 mM LiCl for 24 h and a VEGF ELISA was performed using conditioned cell culture medium. No differences in the relative VEGF levels were detected between untreated controls (1.0±0.02), NaCl- (0.99±0.03) or LiCl-treated cells (1.01±0.02; n≥15 of 2 independent experiments).

### Enhanced Retinal Wnt/β-catenin Signaling Induced by LiCl or SB 216763 Treatment has No Influence on Vessel Regrowth Following an Oxygen-induced Damage

**Figure 5:**
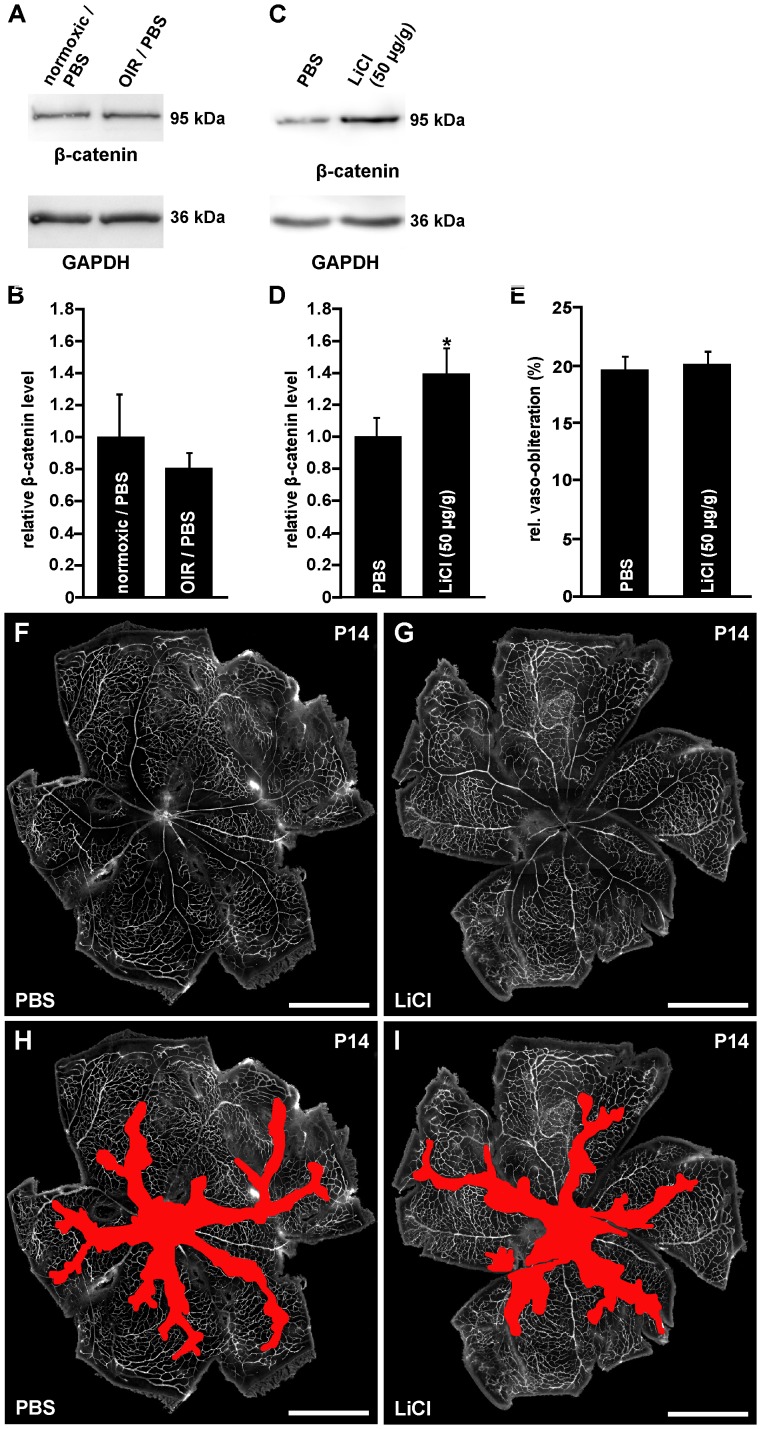
Intraperitoneal LiCl injections activate the Wnt/β-catenin pathway following OIR, but have no influence on vessel regrowth into vaso-obliterated areas. A–D. Western blot analyses (A, C) and relative densitometry (B, D) for β-catenin in retinal proteins of normoxic control FVB/n mice or after exposure to 75% oxygen for five days from P7 and intravitreal PBS injection or i.p. treatment with LiCl at P12. A, B. In retinal proteins from mice 6 h after oxygen exposure and intravitreal PBS injection no difference in β-catenin protein level were detected when compared to normoxic control littermates (mean ± SEM; n = 5 from 2 independent experiments). C, D. In contrast, in LiCl-injected mice a significant increase of β-catenin levels was observed when compared to PBS treated animals (mean ± SEM; n = 13 from 3 independent experiments; *p<0.05). E–I. Representative retinal whole mounts of FVB/n mice that were perfused with FITC-labeled dextran at P14 after induction of OIR and i.p. LiCl (3×50 µg/g body weight, G, I) or PBS injection (F, H). Scale bars: 1000 µm. For quantification the area of vaso-obliteration (red in H, I) was quantified at P14 and plotted as percentage of total area of the superficial vascular plexus (E). No difference in the area of vaso-obliteration was observed between treated mice and control animals (mean ± SEM; n≥13 from 4 independent experiments).

To test if the angiogenic properties of LiCl on cultured HDMEC would increase capillary repair *in vivo* following damage, OIR was induced in mice and vessel regrowth into vaso-obliterated areas was analyzed following treatment with LiCl. First, we analyzed if an induction of an OIR itself alters retinal Wnt/β-catenin signaling. After oxygen exposure for 5 days and additional 6 h after intravitreal PBS injection no difference in retinal β-catenin protein level was observed when compared with normoxic controls (Fig. 5A, B). We next investigated the effect of systemic LiCl administration on the activation of the Wnt/β-catenin signaling pathway in the retina. Following oxygen treatment, a specific band for β-catenin was observed by Western blot analysis of retinal proteins from PBS-injected mice (Fig. 5C). The signal was more pronounced in LiCl-treated mice and densitometry indicated a significant 40% increase in the amounts of β-catenin compared with PBS-injected controls (Fig. 5C, D). To analyze the effect of an i.p. injection of LiCl on vessel regrowth, injected mice were perfused with FITC-labeled dextran and retinal whole-mounts were prepared. At P14 and two days after oxygen treatment of PBS-injected control animals, a central vaso-obliterated area was observed spreading alongside the arteries into the retinal periphery (Fig. 5F and shown in red in Fig. 5H). In LiCl-injected animals, the vaso-obliterated area was not different in morphological appearance or size to that of PBS-injected controls (Fig. 5G and shown in red in Fig. 5I), a finding that was confirmed by quantitative analysis (Fig. 5E).

**Figure 6:**
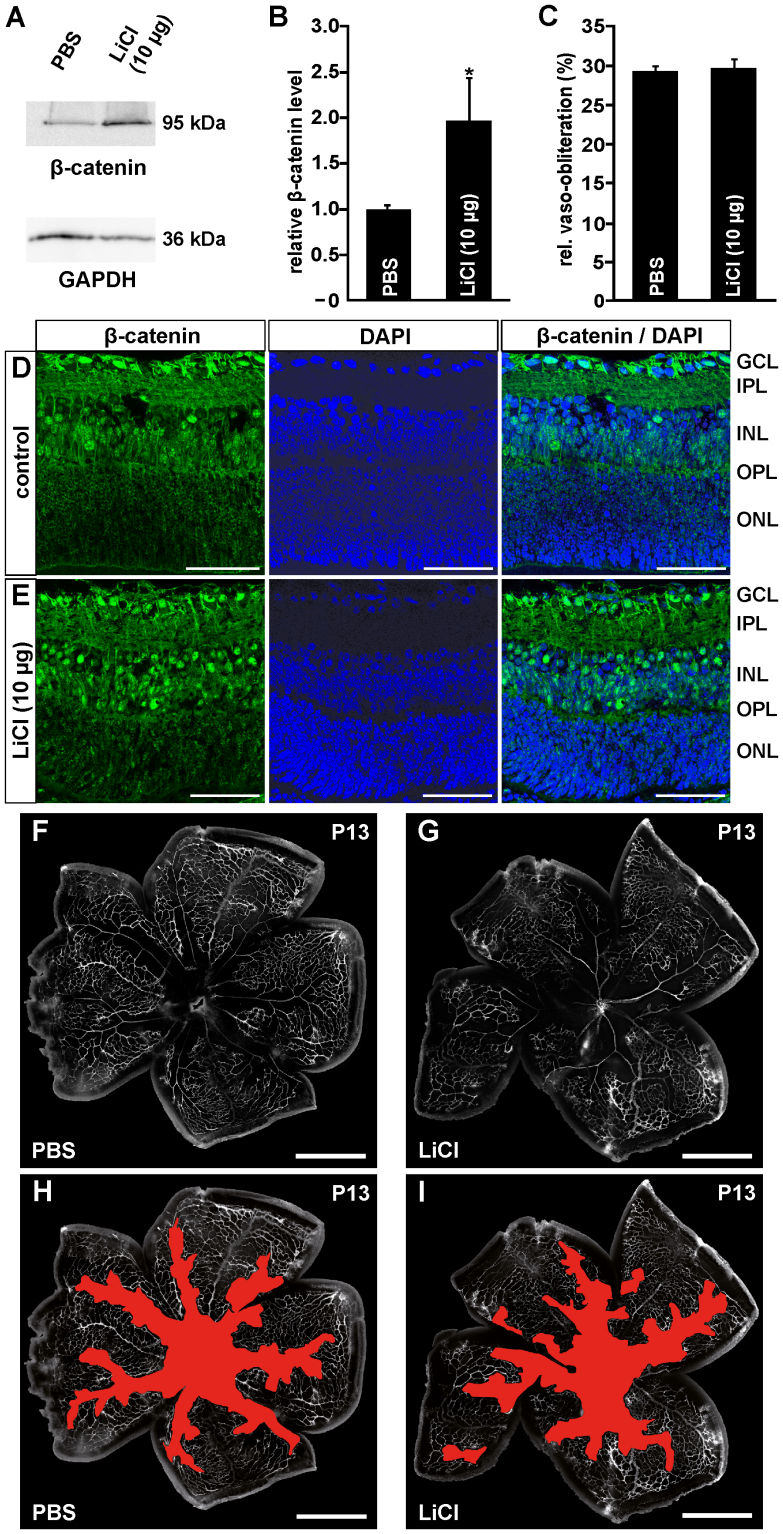
Intravitreal LiCl injections activate the Wnt/β-catenin pathway following OIR, but have no influence on vessel regrowth into vaso-obliterated areas. A, B. Western blot analyses (A) and relative densitometry (B) for β-catenin in retinal proteins of FVB/n mice after exposure to 75% oxygen for five days from P7 and intravitreal application of LiCl (10 µg). After 6 h, in LiCl-injected eyes a significant increase of β-catenin levels was observed compared to PBS treated animals (mean ± SEM; n = 11 from 3 independent experiments; *p<0.05). D, E. Immunohistochemical staining for β-catenin (green) on retinal cryosections from FVB/n mice 6 h after intravitreal LiCl (10 µg) (E) or PBS injection (D) and oxygen-exposure until P12. After treatment with LiCl (E) an intense accumulation of β-catenin is seen in nuclei of the inner nuclear layer compared with PBS injected controls (D). Blue, DAPI staining. Scale bars: 50 µm. F–I. Representative retinal whole mounts of FVB/n mice that were perfused with FITC-labeled dextran at P13 after induction of OIR and intravitreal LiCl (1×10 µg; G, I) or PBS injection (F, H). Scale bars: 1000 µm. For quantification the area of vaso-obliteration (red in H, I) was quantified at P13 and plotted as percentage of total area of the superficial vascular plexus (C). No difference in the area of vaso-obliteration was observed between treated mice and control animals (mean ± SEM; n≥13 from 3 independent experiments).

**Figure 7:**
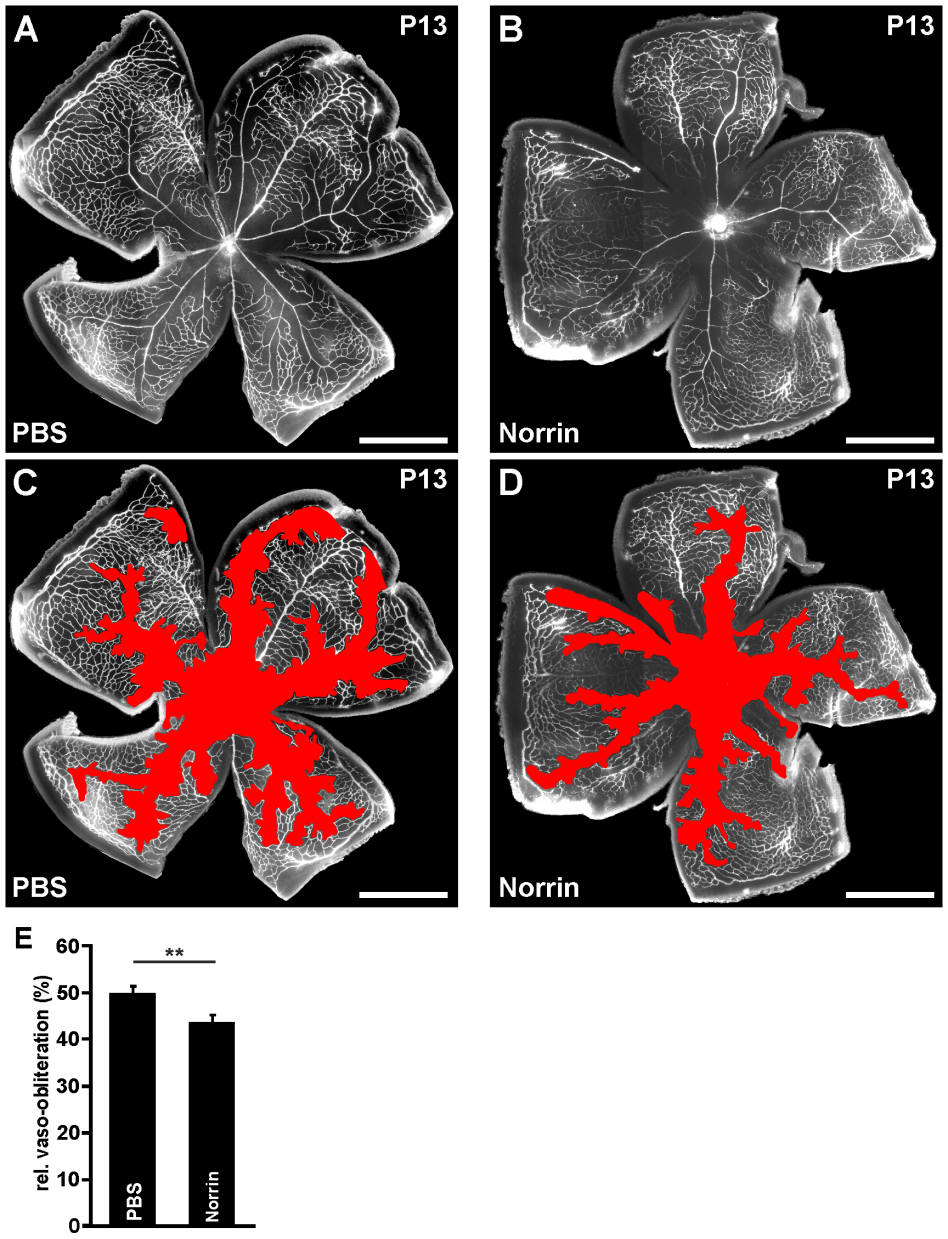
Norrin promotes vessel regrowth into vaso-obliterated areas. A–E. Representative retinal whole mounts of FVB/n mice that were perfused with FITC-labeled dextran at P13 after induction of OIR and intravitreal Norrin (1×3 µl; 10 ng/µl; B, D) or PBS injection (A, C). Scale bars: 1000 µm. For quantification the area of vaso-obliteration (red in C, D) was quantified at P13 and plotted as percentage of total area of the superficial vascular plexus (E). In Norrin-treated mice the area of vaso-obliteration was significantly smaller compared to PBS-injected control animals (mean ± SEM; n = 11 from 3 independent experiments; **p<0.01).

Next we investigated the effects of an intravitreal injection of LiCl. Following oxygen exposure and intravitreal injection of LiCl (10 µg), an approximate 2-fold increase in the levels of β-catenin was detected in retinal proteins compared with control eyes that received PBS only (Fig. 6A, B). We now analyzed by immunohistochemistry whether the increase of total β-catenin in retinal proteins lead to an increase of its presence in retinal nuclei. After induction of an OIR in PBS-injected control eyes, a distinct signal for β-catenin was detected in the nuclei of the retinal ganglion cell layer (GCL), which was weaker in the nuclei of the inner nuclear (INL) layer (Fig. 6D). In eyes that were intravitreally injected with LiCl, the immunoreactivity for β-catenin in the GCL was similar to that of control retinae (Fig. 6E). In contrast to PBS-injected control animals, LiCl-treated mice showed a substantial increase in the number of β-catenin-positive nuclei in the inner nuclear layer (Fig. 6E). Still, the increase in the amounts of β-catenin did not improve regrowth of capillaries following OIR. In both PBS- (Fig. 6F, H) and LiCl-injected eyes (Fig. 6G, I), the morphological appearances and sizes of the vaso-obliterated areas were entirely similar at P13 (Fig. 6C, F–I). For positive control and since Norrin has the potential to induce vessel regrowth into vaso-obliterated areas after oxygen treatment, Norrin (10 ng/ml) or PBS were injected into the vitreous cavity following an OIR at P12. In Norrin-treated eyes the vaso-obliterated area was 6% smaller at P13 compared to PBS-injected control eyes (Fig. 7A–D), a difference that was statistically significant (p<0.01; Fig. 7E).

**Figure 8:**
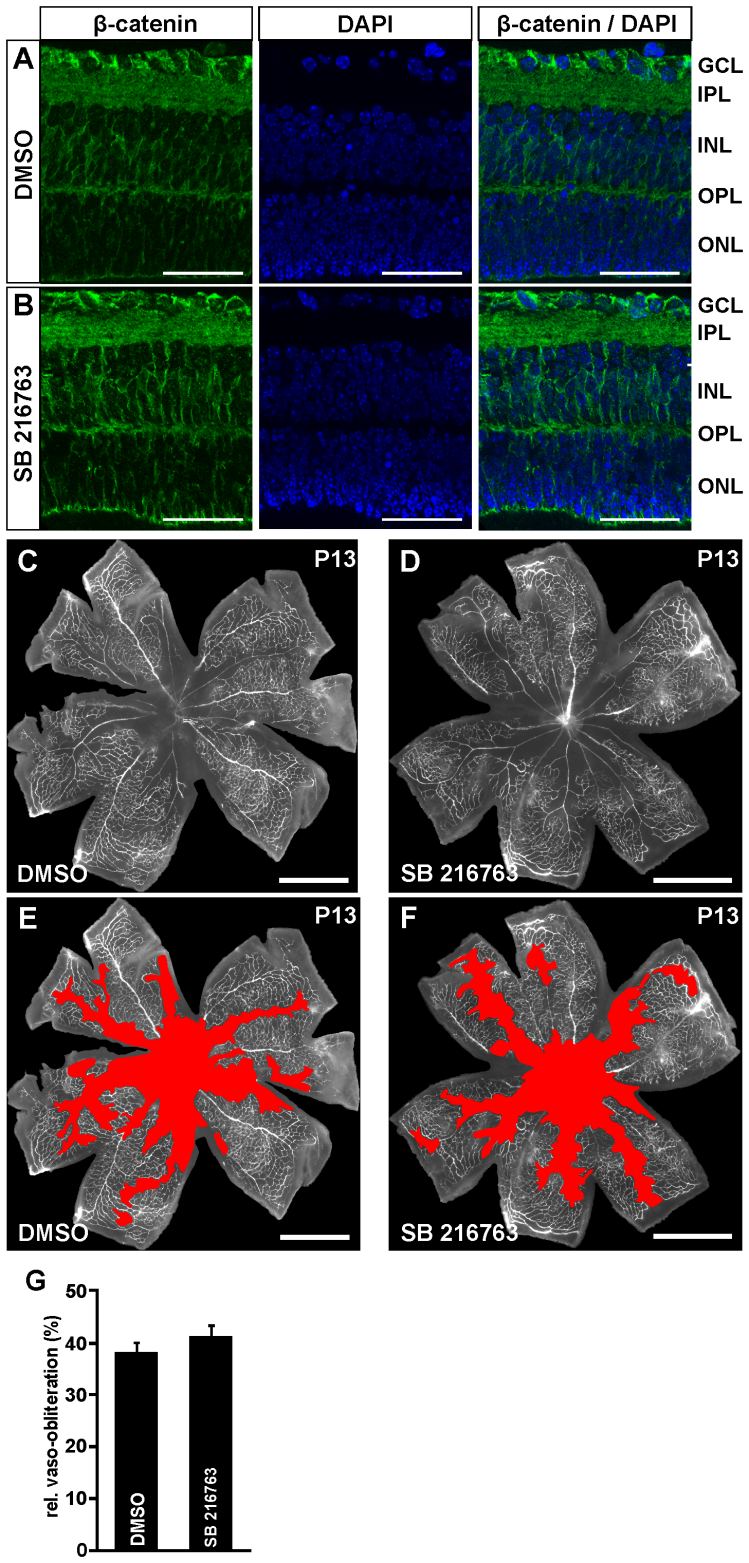
Intravitreal SB 216763 injections activate the Wnt/β-catenin pathway following OIR, but have no influence on vessel regrowth into vaso-obliterated areas. A, B. Immunohistochemical staining for β-catenin (green) on retinal cryosections from FVB/n mice 6 h after intravitreal injection of 3 µl SB 216763 (5 µg/µl) (B) or DMSO injection (A) and oxygen-exposure until P12. After treatment with SB 216763 (B) a moderate accumulation of β-catenin is seen in the inner retina compared with DMSO injected controls (A). Blue, DAPI staining. Scale bars: 50 µm. C–F. Representative retinal whole mounts of FVB/n mice that were perfused with FITC-labeled dextran at P13 after induction of OIR and intravitreal SB 216763 (1×3 µl; 5 µg/µl; D, F) or PBS injection (C, E). Scale bars: 1000 µm. For quantification the area of vaso-obliteration (red in E, F) was quantified at P13 and plotted as percentage of total area of the superficial vascular plexus (G). No difference in the area of vaso-obliteration was observed between treated mice and control animals (mean ± SEM; n = 6 from 2 independent experiments).

Finally, to rule out the possibility that the lack of vessel regrowth after induction of an OIR and activation of retinal Wnt/β-catenin signaling by LiCl is due to potential side effects of LiCl, SB 216763 was injected into the vitreous cavity after oxygen treatment. First we analyzed by immunohistochemistry the potential of SB 216763 to activate Wnt/β-catenin signaling in the retina. Six hours after SB 216763 injection and termination of oxygen treatment a moderate increase of β-catenin staining was observed in the inner retina compared with DMSO-treated controls (Fig. 8A, B). Quite noteworthy, the activation of Wnt/β-catenin signaling by SB 216763 treatment following OIR did again not enhance vessel regrowth into vaso-obliterated areas. Morphology and sizes of the vaso-obliterated areas of SB 216763 injected eyes were entirely similar to that of PBS-treated controls (Fig. 8C–G).

## Discussion

We conclude that treatment with LiCl mediates angiogenic properties on cultured HDMEC via an activation of β-catenin signaling, but not *in vivo* following OIR in the mouse eye. Our conclusions rest upon (1) the capability of LiCl to activate β-catenin signaling in cultured HDMEC and in retinal cells *in vivo*; (2) the finding that LiCl induces proliferation, migration and survival in HDMEC and (3) the observation that a LiCl-mediated retinal activation of β-catenin signaling has no influence on vascular repair following OIR.

The capability to induce proliferation, migration and survival of microvascular endothelial cells *in vitro* are characteristically seen with agents or molecules that have potent angiogenic properties *in vivo* such as VEGF [29], [30]. The comparable effects of LiCl and SB 216763 are most likely mediated via an activation of Wnt/β-catenin signaling since increased levels of β-catenin were observed after treatment of the cells, while quercetin, an inhibitor of Wnt/β-catenin signaling, blocked the effects of LiCl. However, higher concentrations of LiCl led to reduced β-catenin levels in HDMEC and a loss of LiCl-mediated proliferation and viability. Since LiCl at a concentration of 10 mM has the distinct potential to inhibit the enzymatic GSK-3β activity *in vitro* [31], the effects of higher LiCl concentrations on cultured microvascular endothelial cells are most likely mediated via activation or inhibition of additional signaling pathways that inhibit Wnt/β-catenin signaling. In microvascular endothelial cells, the activation of Wnt/β-catenin signaling is supposed to increase VEGF levels after 24 h [32]. However, in our current study we did not observe increased VEGF concentrations in conditioned cell culture medium after treatment of HDMEC with 0.2 mM LiCl for 24 h. In line with our observations, Guo and coworkers described an enhanced expression of VEGF in brain endothelial cells only after incubation with very high concentrations of LiCl such as 10 or more mM [33]. Since we observed the main effects of LiCl on proliferation and migration of HDMEC after treatment with concentrations of 0.2 or 1 mM and after incubation for 24 h or less, our data strongly suggest that those effects are independent from VEGF signaling.

There are several explanations for the discrepancy between the *in vivo* and *in vitro* findings of our study. First, we might have missed to administer LiCl or SB 216763 at the most potent concentration following i.p. or intravitreal injection. We regard this possibility as unlikely as we observed an accumulation of β-catenin in retinal proteins at amounts similar to those observed in a previous study following the intravitreal injection of Norrin [34]. In line, after treatment of mice with the same concentrations of Norrin we found an enhanced vessel regrowth into vaso-obliterated areas.

Another possibility relates to the lack of specificity of LiCl or SB 216763. LiCl may act on neuronal signal transmission and neurotransmitter release through disturbing the homeostasis of sodium, magnesium and calcium [35]. In addition, LiCl influences a broad array of transcription factors and intracellular signaling pathways, in particular that of phosphoinositide 3-kinase, protein phosphatase 2A and monophosphatase [25], [35]. Similar observations were made for SB 216763 that was shown to additionally block various protein kinases such as ERK8, CDK2-CyclinA, DYRK1A or HIPK2 [36]. Also the primary target of LiCl and SB 216763 to cause an accumulation of β-catenin, GSK-3β, has the distinct potential to affect proliferation, migration, apoptosis or survival, and differentiation of various subsets of cell types, via interaction with various transcription factors, metabolic enzymes and signaling proteins [37], [38], [39]. Some of these pathways might have interfered with an *in vivo* angiogenic activity of LiCl or SB 216763 induced via GSK-3β inhibition and β-catenin accumulation.

Finally, both inhibitors of GSK-3β enhanced β-catenin levels, but failed to induce vessel regrowth into vaso-obliterated areas whereas treatment with Norrin improved vascular repair. It is tempting to speculate that an accumulation of β-catenin and the increase in activity of canonical Wnt/β-catenin signaling is essential, but not sufficient to induce retinal capillary repair *in vivo*. In previous work, we could show that inhibition of canonical Wnt/β-catenin signaling by treatment with dickkopf-1 attenuated the effects of Norrin on capillary repair after OIR indicating that this pathway is essential for capillary repair [16]. Still, other and as of yet unidentified pathways might be directly or indirectly activated by Norrin to facilitate its effects on capillary repair. Similar pathways might be also involved in the effects of Norrin on retinal angiogenesis during the development of retinal capillaries and appear to be a very interesting target for further studies.
